# Adult embryonal rhabdomyosarcoma (botryoid subtype) of the ureter: A case report

**DOI:** 10.1016/j.eucr.2026.103400

**Published:** 2026-03-04

**Authors:** Logan Wesemann, Douglas Fair, Kenil Mehta, Glen A. Lau

**Affiliations:** a Rocky Vista University College of Osteopathic Medicine, Ivins, UT, 84738, USA; b Department of Pediatrics, Division of Pediatric Hematology/Oncology, University of Utah, Salt Lake City, UT, 84108, USA; c Huntsman Cancer Institute, Salt Lake City, UT, 84112, USA; d Primary Children's Hospital, Intermountain Healthcare, Salt Lake City, UT, 84113, USA; e Rocky Vista University–Montana College of Osteopathic Medicine, Billings, MT, 59106, USA; f Department of Urology, Division of Pediatric Urology, University of Utah, Salt Lake City UT, 84108, USA

**Keywords:** Embryonal rhabdomyosarcoma, Botryoid rhabdomyosarcoma, Ureter

## Abstract

Embryonal rhabdomyosarcoma arising from the ureter is exceedingly rare. We report the case of a 20-year-old female who presented with flank pain and hematuria, initially attributed to ureteral stones. Subsequent evaluation revealed an embryonal rhabdomyosarcoma (botryoid subtype) of the ureter. Following surgical resection and chemotherapy, the patient achieved remission and experienced minor complications including neuropathy and bladder irritation. This case highlights the classification and management of ureteral rhabdomyosarcoma. While the prognosis for ureteral rhabdomyosarcoma appears favorable based on limited literature, long-term surveillance is crucial for monitoring recurrence and managing potential complications.

## 1 Background

Rhabdomyosarcoma (RMS) is a malignant soft tissue tumor characterized by the presence of skeletal muscle differentiation, predominantly diagnosed in children and adolescents. It is the most common soft tissue sarcoma in the pediatric population, representing roughly 50% of pediatric soft tissue sarcomas. In adolescents and young adults, it is less common, representing less than 10% of the soft tissue sarcomas in this group^1^, ^2^, ^3^. Rhabdomyosarcoma is classified into several histological and molecular subtypes, with the major categories being embryonal, alveolar, spindle cell/sclerosing, and pleomorphic rhabdomyosarcoma. Embryonal rhabdomyosarcoma is associated with the lack of a poor prognostic translocation (PAX 3/7-FOXO1). One subtype of embryonal rhabdomyosarcoma is a botryoid variant which is characterized as “grapelike” polypoid masses with predilection for mucosa-lined, hollow organs such as vagina, bladder, cervix, and, more rarely, the biliary tract and ear^1^, ^2^, ^3^, ^4^, ^5^, ^6^, ^7^. And while embryonal rhabdomyosarcoma of the genitourinary tract is not uncommon,^8^ rhabdomyosarcoma arising from the ureter is extremely rare, with only a few isolated cases reported in the literature^9^, ^10^, ^11^. We present the case and outcome of a 20-year-old female with embryonal botryoid rhabdomyosarcoma of the ureter, including the presentation, management and long-term outcome after treatment.

## 2 Case presentation

### 2.1 Initial presentation and diagnostic evaluation

**Fig. 1:**
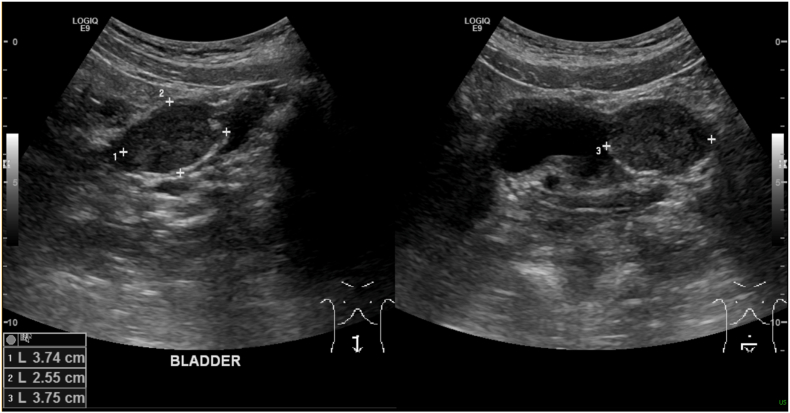
Legend: Transabdominal bladder ultrasonography demonstrating a well-defined, heterogeneous mass measuring 3.8 x 3.7 × 2.6 cm is noted at the left ureterovesical junction. The lesion was initially suspicious for an intravesical thrombus. Associated left-sided hydroureter is visualized superior to the mass.

**Fig. 2:**
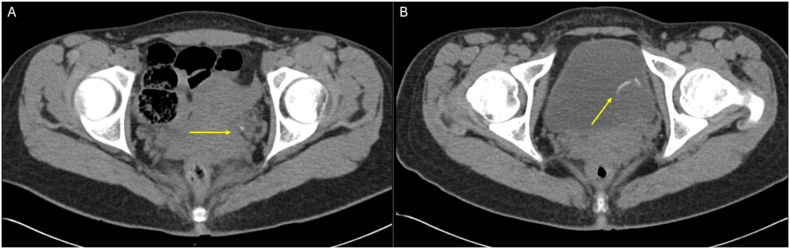
Legend: (A) Initial axial CT scan of abdomen and pelvis without contrast demonstrated moderate hydroureter tapering to a focal calcification in the distal ureter, originally interpreted as an obstructing calculus. (B) Follow-up CT scan without contrast at two months demonstrating progression of a heterogeneous mass. Note the presence of internal macroscopic fat and peripheral calcifications, suggestive of the osseous components within the botryoid rhabdomyosarcoma.

A 20-year-old Caucasian female presented to the emergency department with intermittent left-sided flank pain, hematuria, urinary frequency, and urgency. Initial CT revealed distal left ureteral calcifications with hydronephrosis. Presuming this was a ureteral stone, she was given medical expulsive therapy and discharged home. Two months later, she returned with similar symptoms in addition to left lower quadrant abdominal pain. She reported increased pain and nausea compared to her previous encounter, in addition to passage of large blood clots in her urine over the previous two days. Shown in Fig. 1, a Bladder ultrasound showed hydroureter and a mass measuring 3.8 x 3.7 x 2.6 cm, initially perceived as a thrombus, prompting a repeat CT scan. The repeat CT revealed worsened left-sided hydronephrosis and a new hypoechoic, well-defined ovoid lesion to the left of the bladder with internal calcifications, which was thought to possibly represent a ureterocele. The distal ureter could not be visualized. Fig. 2 shows comparative CT imaging demonstrating significant progression in tumor size with increased calcifications. As the definitive characteristics of the mass could not be established, cystoscopy was performed.

Cystoscopy revealed a non-cystic, non-penetrable lesion arising from the left ureteral orifice, with no evidence of a ureterocele. A retrograde pyelogram showed the distal 6 cm of the ureter filled with tumor. A biopsy was performed, and a ureteral stent was placed to maintain patency in anticipation of a subsequent surgical resection.

### 2.2 Histologic findings

Histologic analysis of the biopsy revealed spindle cell stromal proliferation with cells showing scant cytoplasm, nuclear enlargement, and increased mitotic activity, indicating a malignant process. The immunohistochemical stains for Desmin and Myogenin were positive for both markers. PAX3/7-FOXO1 testing was negative. Taken together, the diagnosis of embryonal rhabdomyosarcoma, botryoid subtype, was rendered. Margins were positive for tumor. A staging CT scan of the chest showed no evidence of metastasis.

In North America, pediatric rhabdomyosarcoma guidelines rely on the Children's Oncology Group (COG) risk stratification. This is based on a combination of TNM Pre-Treatment Staging Classification and Clinical Grouping Classification, and more recently histological subtype and/or FOXO1 fusion status and age at diagnosis for Risk Stratification.^12^ Under the TNM system (based on primary tumor site, size, regional lymph node status and distant metastatic involvement), the ureter is not explicitly categorized. Localized tumors that are less than 5 cm and lymph node negative are either Stage 1 or Stage 2 based on location of tumor. Genitourinary (GU) tumors that are not arising from the bladder or prostate are Stage 1 and GU tumors arising from the bladder or prostate are Stage 2^12^.

### 2.3 Therapeutic intervention and risk stratification

Following multidisciplinary review, the patient underwent robotic distal ureterectomy (7 cm excision with a bladder cuff), pelvic lymph node dissection, and psoas hitch ureteroneocystostomy. A mid-ureteral psoas hitch and peritoneal flap for bladder closure reinforcement were utilized to ensure a tension-free anastomosis. Pathology of resected ureter confirmed a small (<5 cm) embryonal rhabdomyosarcoma (RMS), botryoid subtype, with R0 negative margins and no nodal involvement (T1aN0M0). Based on the complete resection of the localized disease in an R0 manner before starting chemotherapy, and its proximity to bladder, the patient was classified as Stage 2, Clinical Group I.

Postoperatively, imaging confirmed no evidence of disease or anastomotic leak. After fertility preservation (embryo cryopreservation with her partner), the patient received 14 cycles of chemotherapy, which were alternating cycles of VAC (Vincristine, Dactinomycin, and Cyclophosphamide) with VI (Vincristine and Irinotecan) of adjuvant therapy according to COG protocol ARST1431 Arm A (no maintenance therapy). We did not include radiation for local control because of the R0, negative resection, Clinical Group I status. The patient tolerated the therapy well, experiencing mild flank pain and mild reversible neuropathy during therapy. After completion of therapy, the patient was surveyed with MRI of the abdomen and pelvis and CXR every 3 months for the first two years off of therapy, and then every six months from years three to five. The patient is now greater than 5 years post-therapy and remains disease-free.

## 3 Discussion

This report is unique for several reasons. The number of currently reported cases is three; thus, this case adds to the limited understanding of the presentation, clinical course, and treatment of ureteral RMS, highlighting the rarity of this diagnosis. In contrast to previously reported cases^9^, ^10^, ^11^—where patients neither exhibited early ureteral stone symptoms nor had a significant history of nephrolithiasis—our patient presented with renal colic and concern for obstructing ureteral calculi. This presentation likely indicates early ureteral obstruction due to tumor involvement of the distal ureteral wall, either with calcifications in the tumor or small ureteral stones, whose passage was impeded by the tumor. The absence of similar findings in earlier cases underscores the unique diagnostic challenge posed by our patient's presentation.

Across all reported cases, including ours, common symptoms mimicked those of ureteral obstruction, including flank pain, nausea, vomiting, and hematuria (see Table 1). Imaging consistently revealed hydronephrosis and/or hydroureter, underscoring the importance of maintaining a broad differential diagnosis in young patients with persistent obstructive uropathy and atypical presentations.

**Table 1:** Summary of previously reported ureteral rhabdomyosarcomas and our case.

Case year	Age	Sex	Clinical Symptoms	Imaging Findings (side)	Staging	Treatment	Disease Status	Learning Points
1999	19	F	Flank pain, nausea, vomiting	Hydronephrosis (R)	T2aN0M0 CG I	Surgical resection, Chemotherapy, and radiation	Disease-free 3 years post treatment	Multimodal therapy increases 5-year survival
2008	4	F	Not discussed	Hydronephrosis (L)	T2aN0M0 CG III	Chemotherapy, radiation, followed by surgical resection	Not discussed	Consider initial chemotherapy or radiation to shrink tumor (12 weeks)
2023	29	F	Flank pain, hematuria, hypertension	Hydronephrosis, hydroureter (R)	T2aN0M0 CG I	Chemotherapy followed by surgical resection	Disease-free 18 months post treatment	Surgery and chemotherapy are effective for adult patients Ongoing post-treatment surveillance is crucial
2025 (this case)	*20*	*F*	*Flank pain, hematuria, frequency, urgency*	*Hydronephrosis, hydroureter (L)*	T2aN0M0 *CG I*	*Surgical resection followed by chemotherapy*	*Disease-free 4-years post treatment*	*Ureteral RMS may be a favorable site* *Establishing formal criteria for ureter may help better risk stratify in the future*

All embryonal botryoid subtype cases currently reported in literature, including our case. CG = Clinical Group.

Our patient did well following surgical resection and chemotherapy, apart from occasional bladder irritation episodes and chemotherapy-induced neuropathic symptoms. All cases appear to have resulted in the successful treatment of the disease, although the long-term sequelae of ureteral RMS has not been described in the literature. Interestingly, all four documented cases have occurred in female patients.

### 3.1 Diagnosis and treatment of ureteral RMS

Following biopsy, assessment of suspected RMS should include axial imaging for staging and group assignment (after initial surgery resection) and with histological and/or molecular testing, Risk assessment should be made, which can guide chemotherapy and other adjuvant therapy. Further management described in the literature emphasizes multimodal therapy, but the sequence of this is variable in the described cases.

One case of an adult female who presented with one week of constant, dull, right flank pain, associated with intermittent nausea and vomiting. CT urography showed right hydronephrosis as well as a distal ureter filling defect. Biopsy revealed the definitive diagnosis of embryonal RMS botryoid subtype. They performed a nephroureterectomy due to possible tumor seeding from previously stenting the patient. They concluded by mentioning the value of multimodal therapy to increase the 5-year survival rate in patients with RMS.^9^

The next case involved a 29-year-old pregnant female who presented with right flank pain, hematuria, and hypertension with a urinalysis positive for blood and leukocytes and a significant past medical history of kidney stones. Ultrasound imaging discovered unilateral right hydronephrosis and right hydroureter. Post-partum, the patient's symptoms persisted, after which a CT urogram revealed a thick-walled enhancing segment of the right proximal ureter, which proved to be RMS. After initial chemotherapy, the distal ureterectomy was performed. The authors concluded that surgical intervention and chemotherapy are effective treatment options for patients in this category and should be followed by ongoing post-treatment surveillance for oncologic control.^10^

The last case involved a 4-year-old female with a larger 8.0 x 4.0 x 2.0 cm RMS initially thought to be originating at the bladder trigone. Biopsy showed a botryoid subtype of RMS. The patient was first treated with chemotherapy and radiation (likely due to larger tumor size), after which the tumor was grossly resected. Gross resection revealed positive margins at the ureter and vagina. They concluded by describing the use of tumor, node, metastasis (TNM) staging to determine proper diagnosis and treatment.^11^

### 3.2 Surgical approach and technique consideration

In this case, a distal ureterectomy with a psoas-hitch ureteroneocystostomy was performed to achieve a tension-free anastomosis. Minimizing tension is fundamental to optimizing healing and reducing the risk of stricture or anastomotic failure.^13^ For distal defects, bladder mobilization with or without a psoas hitch facilitates this objective by reducing the distance between the viable ureteral stump and the bladder. When this mobilization is insufficient to achieve a tension-free repair, a Boari flap can be employed as an adjunct technique.^14^ For more proximal or extensive ureteral loss, options include transureteroureterostomy or ileal ureter substitution, while mid-ureteral lesions may require a Boari flap with psoas hitch. Regardless of technique, the primary goal is a tension-free, well-vascularized anastomosis.^15^

### 3.3 Ureteral rhabdomyosarcoma classification

The classification and treatment of ureteral RMS remain poorly defined in the literature, particularly when compared to other genitourinary (GU) tumors such as bladder and prostate RMS. Molecular markers, such as FOXO1 fusion status, play a crucial role in defining prognosis across all RMS cases. In the case of our young adult female patient, her negative FOXO1 fusion status–a marker typically associated with better outcomes–aligned with her favorable response to treatment. Negative fusion status is increasingly recognized as a prognostic marker, helping to stratify patients into low, intermediate, or high-risk groups, which informs treatment decisions. However, the absence of specific guidance for ureteral RMS presents challenges in how best to incorporate molecular profiling into personalized care.

Additionally, ureteral RMS presents unique challenges in surgical management due to the ureter's proximity to other important structures. Therefore, similar management strategies must be adapted for ureteral tumors, particularly when procedures such as ureteral reimplantation are required. These procedures raise the complexity of treatment and necessitate careful planning to preserve function while ensuring complete tumor resection. Developing a more refined classification for ureteral RMS that acknowledges these surgical challenges will be essential in future guidelines.

Another key issue is the lack of consensus on whether ureteral RMS arises from a "favorable" or "unfavorable" site. The lack of a standardized classification for ureteral rhabdomyosarcoma (RMS) complicates the development of a unified treatment approach. However, excluding one pediatric case with unknown follow-up, reported cases, including ours, demonstrate favorable outcomes. This trend likely reflects early detection, as symptomatic ureteral obstruction from even minimal tumor growth may be prompting clinical evaluation before distant metastasis. This consistent pattern suggests that ureteral RMS might be considered a favorable site. Establishing formal criteria to define ureteral RMS as arising from either a favorable or unfavorable site could improve future risk stratification and guide management decisions.

## 4 Conclusion

This case highlights the diagnostic challenges of ureteral botryoid rhabdomyosarcoma, a rare malignancy that often mimics common obstructive uropathy. Our patient's five-year disease-free survival underscores the efficacy of multimodal therapy combining radical resection with risk-adapted chemotherapy. However, the lack of a standardized COG classification for the ureter remains a significant gap in oncologic frameworks. Given the favorable outcomes reported here and in existing literature, we propose formalizing the ureter as a "favorable" primary site. Ultimately, a high index of suspicion for atypical obstructive symptoms in young patients is essential for early detection and the refinement of future risk-stratification protocols is warranted.

## CRediT authorship contribution statement

**Logan Wesemann:** Writing – review & editing, Writing – original draft, Methodology, Investigation, Conceptualization. **Douglas Fair:** Writing – review & editing, Writing – original draft, Validation, Supervision. **Kenil Mehta:** Writing – review & editing, Writing – original draft, Investigation. **Glen A. Lau:** Writing – review & editing, Writing – original draft, Validation, Supervision.

## Funding

Funding was provided by Rocky Vista University College of Osteopathic Medicine, 255 E Center St, Ivins, UT, 84738, USA.
